# Serum test for secretory component-containing anti-citrullinated protein antibodies as a novel prognostic tool in rheumatoid arthritis at-risk subjects

**DOI:** 10.1016/j.jtauto.2025.100317

**Published:** 2025-09-17

**Authors:** Klara Martinsson, Simon Åhammar, Alexandra Cîrciumaru, Bence Réthi, Michael Ziegelasch, Aase Hensvold, Alf Kastbom

**Affiliations:** a Division of Inflammation and Infection, Department of Biomedical and Clinical Sciences, Linköping University, Sweden; b Clinical Department of Rheumatology in Östergötland, Region Östergötland, Linköping, Sweden; c Division of Rheumatology, Department of Medicine Solna, Karolinska Institutet, Stockholm, Sweden; d Center for Rheumatology, Academic Specialist Center, Stockholm Health Services, Region Stockholm, Sweden

**Keywords:** Rheumatoid arthritis, Prognostic value, Testing, Secretory component-containing anti-citrullinated protein antibodies

## Abstract

Individuals with autoantibodies against citrullinated proteins (ACPA) and musculoskeletal symptoms face increased risk of developing rheumatoid arthritis (RA). There are knowledge gaps concerning who will develop disease, and predictors of RA onset are highly warranted. A mucosal origin hypothesis in RA is gaining increasing support, for instance by a previous study showing that secretory component-containing ACPA (SC ACPA) in the circulation are prognostic for RA onset. This study aimed to confirm the prognostic value of serum SC ACPA in a large cohort of symptomatic at-risk subjects and to establish a cutoff level for the prognostic use of SC ACPA testing.

Baseline sera from an observational prospective cohort of IgG ACPA positive individuals with musculoskeletal complaints (Karolinska cohort, n = 266) were tested for SC ACPA by ELISA. SC ACPA levels were increased among subjects subsequently developing arthritis (n = 100, median 62 arbitrary units (AU)/mL) compared to those who did not (n = 166, median 40 AU/mL; p < 0.001). A cutoff level for the optimal discrimination concerning future arthritis onset was established by Youden's index, resulting in 81 subjects (30 %) testing positive for SC ACPA, whereof 45 (56 %) progressed to arthritis. Among those testing negative (n = 185), significantly fewer progressed (n = 55, 30 %; p < 0.001). This cutoff was then tested in the previously studied at-risk cohort (n = 82), revealing similar prognostic performance in both cohorts (sensitivity 45 % and 41 %; specificity 78 % and 81 %). We conclude that serum SC ACPA testing is of potential clinical value in symptomatic at-risk subjects and strengthens the mucosal association in RA development.

## 1 Introduction

The mucosal immune system has repeatedly been suggested to play a role in early phases of rheumatoid arthritis (RA) development [1,2]. Therefore, important prognostic information may potentially be derived from studying mucosal immune processes in subjects prior to RA onset, *e.g.* prediction of which individuals who will eventually develop disease. Secretory component (SC)-containing antibodies are normally formed at mucosal surfaces, where SC becomes attached to dimeric IgA or IgM during transepithelial antibody transport [3]. We and others previously reported that RA-associated autoantibodies containing SC can be found in the circulation [4,5]. We also showed that serum levels of SC-containing anti-citrullinated protein antibodies (SC ACPA) correlate with levels found locally in the oral cavity and in the lungs [6,7], implying that serum levels at least partly reflect mucosal antibody production. SC ACPA in serum is found in around 20 % of early RA patients, of whom practically all also test positive for IgG ACPA [4].

Patients with IgG ACPA and musculoskeletal complaints (MSK-C) face greatly increased risk of arthritis development, with progression rates ranging from 20 % to 60 % (in median 32 %) within the following few years [8]. The progression rate is profoundly influenced by autoantibodies; IgG ACPA levels, occurrence of ACPA reactivities, as well as concurrent presence of anti-carbamylated protein antibodies and/or rheumatoid factor (RF) [[9], [10], [11]]. To a lesser degree, the progression rate is also influenced by symptom characteristics, since severe inflammatory type of pain in hands and feet is associated with higher risk estimates than populations recruited based on unspecific definitions of musculoskeletal symptoms [11]. Nevertheless, currently clinical available prognostic tools are insufficient for rheumatologists to accurately determine which patients will progress to RA, nor the timing when this occurs. Such knowledge would significantly pave the way for personalized medicine and improve clinical management of at-risk individuals.

We previously reported that serum levels of SC ACPA were prognostic for arthritis development among individuals with IgG ACPA and MSK-C, also after adjusting for levels of IgG ACPA and RF [12], *i.e.* the autoantibodies currently used in clinical practice. However, the finding was not investigated in an independent cohort, and no cutoff level optimized for the prediction of arthritis onset among IgG ACPA positive subjects was established. Therefore, we set out to confirm the prognostic value of serum SC ACPA testing in a large independent cohort. Furthermore, we aimed to establish an SC ACPA cutoff level optimized for prognostic use in IgG ACPA positive symptomatic at-risk individuals, and to test this cutoff level in the cohort where the original finding was made.

## 2 Cohorts and methods

### 2.1 Research individuals

We studied two independent and geographically distinct prospective observational cohorts of ACPA-positive at-risk individuals with musculoskeletal complaints (MSK-C) from Sweden. The Karolinska risk RA cohort recruited patients from the Stockholm area, while the TIRx cohort, where the original finding concerning SC ACPA levels versus progression was made, enrolled patients from the Linköping area in Southeastern Sweden [12]. The Karolinska risk RA cohort enroll individuals with MSK-C referred to the rheumatology clinic being positive for 2nd generation anti-cyclic citrullinated peptide antibodies and at inclusion lack clinical and ultrasound-detected synovitis [8]. Individuals are followed for at least three years, or until progression to clinically manifest arthritis. The current study includes data and baseline serum samples from 266 individuals enrolled between May 2014 and April 2019. Median follow-up was 49 months (IQR: 22–60). Arthritis diagnosis was defined clinically by a trained rheumatologist (joint swelling at physical examination) and in the vast majority the joint swelling was verified by ultrasound. At arthritis onset 91 (90 %) individuals had classifiable RA according to the 2010 criteria and 85 (84 %) initiated disease-modifying-anti-rheumatic-drugs (DMARD).

The Linköping TIRx cohort enrolled 82 IgG ACPA positive individuals with MSK-C who at inclusion lacked clinical synovitis (joint swelling at physical examination) [9]. In contrast to the Karolinska risk RA cohort, ultrasound-detected synovitis was not an exclusion criterion. Median follow-up of the individuals was 53 months (IQR: 9–71). At arthritis onset, 38 (97 %) individuals had classifiable RA according to the 2010 criteria and 28 (72 %) initiated DMARD. 100 blood donor controls, age-matched with TIRx, 50 % women, mean age 52 years, were also included.

The study is conducted in accordance with the Declaration of Helsinki, with approval from the Swedish Ethical Review Authority, ethics approval number: 2022-05779-02 and 2018-2468-31-2. All study participants provided written informed consent.

### 2.2 Autoantibody analyses

In both cohorts, IgG Anti-CCP2 and IgM RF were analyzed at inclusion in a routine clinical laboratory and were defined as positive if above upper limit of normal according to the clinical laboratory. *RF level was reported in international units (IU/ml), and an international standardized cut-off was utilized to define positivity and negativity at each of the accredited Clinical Immunology laboratories affiliated to the participating hospitals.*

SC ACPA was measured in baseline sera as previously described [12], *i.e.*by utilizing commercially available Anti-CCP2 ELISA kits (Euro-Diagnostica, Malmö, Sweden) but with a secondary antibody targeting anti-human SC (Nordic Biosite, Täby, Sweden). The intra- and interassay variability in the SC ACPA ELISA were 3 and 13 %, respectively.

### 2.3 Statistical analysis

SC ACPA levels at baseline were investigated by a receiver operating characteristic (ROC) curve and Youden index. Sensitivity and specificity of elevated SC ACPA for arthritis progression were estimated by 2x2 tables and compared with results from testing of RF in the clinical laboratory. *Kaplan-Meier analysis and log-rank test were performed to analyze the arthritis-free survival time depending on presence of* SC ACPA status *at baseline. Cases (arthritis progressors) were treated as censored at the date of their first visit with arthritis.* SC ACPA status (binary variable), and progression to arthritis were investigated in *univariable and multivariable cox regression analysis.* Statistical analyses were performed with SAS (V.9.3) and SPSS software, and the level of significance was set as 0.05. GraphPad Prism (V 9.1.0) software was used for graphs and images.

## 3 Results

### 3.1 Serum SC ACPA in relation to baseline characteristics and arthritis development

**Fig. 1:**
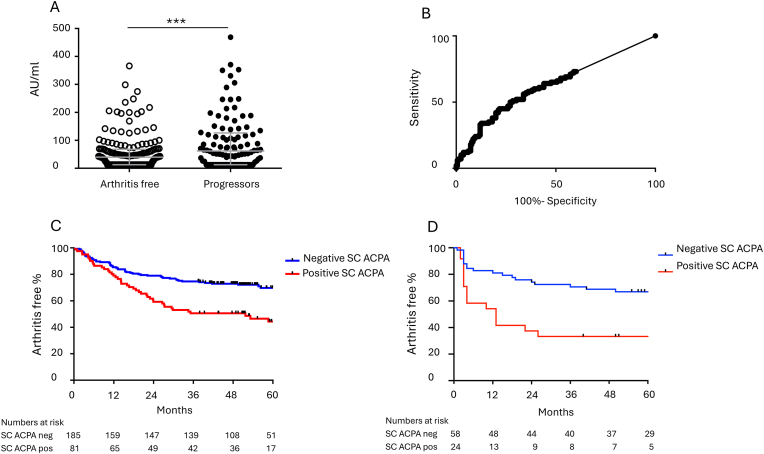
SC ACPA levels in risk RA individuals. A) Baseline secretory (SC) anti-citrullinated protein antibodies (ACPA) levels (AU/ml) in relation to future progression to arthritis in Karolinska risk RA individuals. Line at median with interquartile range. ∗∗∗p < 0.001. B) Receiver operating curve (ROC) for SC ACPA levels and discrimination of individuals progressing to arthritis from individuals remaining arthritis free. Cut off set by Youden index at 70 AU/ml. C) Kaplan–Meier curve showing arthritis-free survival time in Karolinska risk RA individuals in relation to serum SC ACPA status at baseline. D) Kaplan–Meier curve showing arthritis-free survival time in Linköping TIRx individuals in relation to serum SC ACPA status at baseline.

SC ACPA levels were analyzed in 266 baseline samples from Karolinska risk RA individuals. Progression to arthritis was observed in 100 individuals (38 %) after a median of 14 months of follow-up (IQR: 6–27). SC ACPA levels were significantly increased in baseline samples from individuals subsequently progressing to arthritis (median 62 AU/ml, IQR 13–125) compared to individuals remaining arthritis-free during follow up (median 40 AU/ml, IQR 13–67; p < 0.001) (Fig. 1A). SC ACPA levels were analyzed using receiver operating characteristics (ROC) curve to define an optimal cut-off to discriminate individuals progressing to arthritis from non-progressors (Fig. 1B). Optimal cut off for discrimination was set to 70 AU/ml, resulting in 81 patients (30 %) with a positive SC ACPA test, whereof 45 (56 %) progressed to arthritis. Among the patients with a negative SC ACPA test, 55 out of 185 (30 %) progressed to arthritis, which was significantly less common when compared to the SC ACPA positive patients (p < 0.001). The cutoff level of 70 AU/mL corresponded to the 82nd percentile among 100 controls (Supplementary Fig. 1).

Clinical characteristics such as self-reported symptoms, symptom duration, smoking exposure and body mass index were similar between patients with a positive or negative SC ACPA test (Supplementary Table S1). However, SC ACPA positive patients were more often RF positive with higher levels of IgG ACPA (anti-CCP2). Also, SC ACPA positive patients had on average shorter follow-up time reflecting the increased rate of progression.

### 3.2 Serum SC ACPA test as a prognostic factor

We further investigated the prognostic effect of a positive SC ACPA test by survival analysis. Kaplan–Meier curves illustrated increased progression rates among patients with a positive SC ACPA test (>70 AU/mL) in the Karolinska risk RA cohort as well as the TIRx cohort (Fig. 1C + D). This was substantiated in Cox regression analyses, where a positive SC ACPA test was found to be a significantly prognostic in the Karolinska risk RA cohort (HR 2.1 (95 % CI 1.4–3.1; p = 0.0002)), as well as in the TIRx cohort (HR 2.4 (95 % CI 1.3–4.6; p = 0.008)), both in univariable and adjusted analyses (Table 1).

**Table 1:** Elevated SC-ACPA levels as a prognostic factor for arthritis development.

	HR, 95 % CI	p value	∗HR, 95 % CI	p value
Karolinska risk RA	2.1 (1.4–3.1)	**0.0002**	1.8 (1.1–2.9)	**0.02**
TIRx	2.4 (1.3–4.6)	**0.008**	2.3 (1.0–5.3)	**0.047**

Cox regression analysis of elevated SC ACPA and progression to arthritis, in univariable analysis (unadjusted) and adjusted∗, multivariable, analysis including anti-CCP2 levels, RF levels, DAS28 and CRP levels. Abbreviations: HR, hazard ratio; CI, confidence interval.

### 3.3 SC ACPA testing in comparison to RF

RF is, apart from IgG ACPA, the most widely used autoantibody analysis for prognostic purposes in RA at-risk patients. Therefore, we wished to relate the diagnostic accuracy of SC ACPA, using the cutoff established in the Karolinska risk RA cohort, to that of RF test results obtained in clinical routine, *i.e.* from the clinical immunology laboratory. In both cohorts investigated, we found numerically overlapping estimates and confidence intervals of sensitivity, specificity, negative predictive values and positive predictive values between SC ACPA and RF tests (Table 2). Furthermore, SC ACPA positivity remained significantly prognostic for progression in Cox regression analyses within the RF negative group in both the Karolinska risk RA cohort (HR 2.1 95 %CI 1.1–4.0, p = 0.02) and the TIRx cohort (HR 2.8 95 %CI 1.2–6.6, p = 0.02).

**Table 2:** Performance of baseline SC ACPA and RF in relation to arthritis development in two independent at-risk cohorts.

SC ACPA	Sensitivity	Specificity	PPV	NPV
Karolinska risk RA (n = 266)	45 % (35–55)	78 % (72–85)	55 % (45–66)	70 % (64–77)
among RF negative (n = 179)	26 % (14–38)	89 % (84–95)	48 % (29–67)	76 % (69–82)
TIRx (n = 82)	41 % (27–57)	81 % (67–90)	67 % (47–82)	60 % (48–72)
among RF negative (n = 58)	35 % (19–55)	86 % (71–94)	62 % (36–82)	67 % (52–79)


**RF**				

Karolinska risk RA	50 % (40–60)	78 % (71–84)	57 % (47–68)	72 % (65–79)
TIRx	41 % (27–57)	81 % (67–90)	67 % (47–82)	60 % (48–72)

Sensitivity, specificity, positive predictive value, PPV; negative predictive value, NPV, are reported with estimates in percentage and (95 % confidence intervals). Subgroup numbers are to be interpreted with caution due to limited sample sizes resulting in wide confidence intervals.

## 4 Discussion

This study establishes a prognostic value of serum testing for SC ACPA in individuals with IgG ACPA and musculoskeletal complaints. We present a cutoff level above which SC ACPA positivity significantly predicts arthritis onset in a clinically relevant at-risk setting. Although the sensitivity is modest in the two cohorts (45 % and 41 %, respectively), we find the specificity for progression (78 % and 81 %, respectively) sufficient for a potential use in clinical decision-making. In the adjusted analyses, we find that SC ACPA status is significantly prognostic also when taking the currently most used autoantibodies (IgG ACPA and RF) into consideration. The prognostic accuracy of SC ACPA is within the same range as RF in both cohorts studied, and importantly, SC ACPA remains a significant predictor in the large group of RF negative patients. Since progression is considerable also in this IgG ACPA positive/RF negative group (28 % in both cohorts), prognostic tools in this population are of significant value. Taken together, we see potential in SC ACPA testing, alongside RF, to provide clinically valuable information for risk stratification of IgG ACPA–positive symptomatic at-risk individuals. In routine care, this could, for instance, be implemented as SC ACPA and RF reflex tests following IgG ACPA positivity. While this approach could potentially optimize referral procedures and risk stratification of at-risk individuals by rheumatologists, measures should also be taken to avoid repeated testing and to refrain from SC ACPA testing in patients with manifest arthritis, as current data do not support such use [13,14].

The mucosal association of serum SC ACPA remains incompletely understood. One potential source can obviously be entrance of antibodies to the circulation through leaky epithelial surfaces, but active re-transportation mechanisms across the epithelium is suggested to exist for secretory antibodies via the CD71/transferrin receptor in some diseases like celiac disease [15]. We have also previously showed that SC ACPA may form in serum if free SC is present [16] which indeed is the case in pre-RA sera [17]. Nevertheless, our hypothesis is that the mucosa is crucial for SC ACPA formation in the circulation, since SC/poly-IgR is required for this to occur [3], and to our knowledge SC/poly-IgR is only expressed at epithelial sites. Therefore, we find it unlikely that SC ACPA would form independently of events at the mucosa.

The SC ACPA assay we used detects SC-containing ACPA of both IgA (dimeric) and IgM isotypes. Van Delft and colleagues showed that SC ACPA occur of both isotypes, but that IgM is increased compared to IgA in early RA sera [5]. In the at-risk phase, however, the isotype ratio of SC ACPA is not yet known, and whether IgM- or IgA-specific -specific SC ACPA analysis could further improve the prognostic ability is an important task for future work. Given the growing body of evidence for a mucosal involvement in RA development, future interventional strategies to prevent RA may be directed towards the mucosa. In this context, serum SC ACPA could be of interest to evaluate both in relation to treatment outcomes and to symptoms or immune alterations at mucosal sites.

In addition to the lack of isotype-specific SC ACPA analysis, we acknowledge some more limitations of the current study. Analyses of mucosal fluids for ACPA analyses, as well as additional analysis of possible mucosal triggers such as periodontitis or microbial dysbiosis would have been of great interest. Also, although SC ACPA intriguingly can occur in synovial fluid from inflamed RA joints [16], we still lack knowledge about their functional properties and their possible role in triggering arthritis. Finally, as the study was conducted at University Hospital clinics, the possibility of referral bias cannot be entirely excluded. However, in Sweden, IgG ACPA is the recommended serological marker when RA is suspected, and according to national guidelines all newly identified ACPA-positive cases are referred to Rheumatology units. For this specific patient group, the participating hospitals functioned as secondary care centers. Therefore, we consider the risk of referral bias in this study to be limited. Strengths of the study include the use of two independent prospective cohorts with at-risk patients highly representative for those seen in daily practice. Both the proportion of positive patients and the prognostic value of a positive test turned out similar despite that one cohort included patients with subclinical signs of inflammation on ultrasound, while the other did not. Therefore, the results are presumably applicable regardless of the availability of ultrasound examination in the clinical setting. It also suggests that SC ACPA has prognostic value even in the latest at-risk phases, where imaging may reveal signs of inflammation.

### 4.1 Conclusion

Serum analysis of SC ACPA identifies a subset of IgG ACPA positive at-risk individuals who are particularly prone to progress to arthritis. The performance of serum SC ACPA testing for prediction of arthritis onset is similar to that of RF and hence of potential clinical value. Furthermore, these results bring yet another link between mucosal surfaces and RA development.

## CRediT authorship contribution statement

**Klara Martinsson:** Writing – review & editing, Writing – original draft, Project administration, Methodology, Formal analysis, Data curation, Conceptualization. **Simon Åhammar:** Writing – review & editing, Resources, Methodology, Investigation. **Alexandra Cîrciumaru:** Writing – review & editing, Methodology, Investigation. **Bence Réthi:** Writing – review & editing, Resources, Methodology, Conceptualization. **Michael Ziegelasch:** Writing – review & editing, Resources, Methodology. **Aase Hensvold:** Writing – review & editing, Writing – original draft, Supervision, Resources, Project administration, Investigation, Funding acquisition, Formal analysis, Data curation, Conceptualization. **Alf Kastbom:** Writing – review & editing, Writing – original draft, Validation, Supervision, Resources, Project administration, Methodology, Investigation, Funding acquisition, Formal analysis, Data curation, Conceptualization.

## Funding sources

This is work was supported by the 10.13039/501100004359Swedish Research Council/Vetenskapsrådet (2017-00359); the 10.13039/501100010767Innovative Medicines Initiative project RTCure; the ERC grants (772209 PREVENT RA); the Knut and Alice Wallenberg Foundation; the King Gustaf V Foundation; the Research Council of Southeast Sweden (10.13039/100010805FORSS); the ALF-grants from Region Östergötland and the 10.13039/501100007949Swedish Rheumatism Association.

## Declaration of competing interest

Alf Kastbom has received honoraria for educational events from AbbVie, and Michael Ziegelasch has received payment and honoraria for educational events from AbbVie, Janssen, Novartis and UCB.

## Data Availability

The authors do not have permission to share data.
