# Supplementary material for: Serum test for secretory component-containing anti-citrullinated protein antibodies as a novel prognostic tool in rheumatoid arthritis at-risk subjects
Source: J Transl Autoimmun. 2025 Sep 17;11:100317. doi: 10.1016/j.jtauto.2025.100317 (PMC12495228; doi:10.1016/j.jtauto.2025.100317)
Supplement: Multimedia component 1 [file mmc1.docx]

**Supplementary Figure 1**

SC ACPA levels in the two at-risk cohorts (Karolinska risk RA and TIRx) and blood donors. The dotted line indicates the cut off set by Youden index at 70 AU/ml.
