# Supplementary material for: Serum test for secretory component-containing anti-citrullinated protein antibodies as a novel prognostic tool in rheumatoid arthritis at-risk subjects
Source: J Transl Autoimmun. 2025 Sep 17;11:100317. doi: 10.1016/j.jtauto.2025.100317 (PMC12495228; doi:10.1016/j.jtauto.2025.100317)
Supplement: Multimedia component 2 [file mmc2.docx]

**Supplementary Table S1. Characteristics of Karolinska risk RA individuals with/without elevated SC ACPA.**

| Variable | Negative SC ACPA  N= 185 | Positive SC ACPA  N= 81 | P-value |
| --- | --- | --- | --- |
| Age, years, mean (SD) | 47 (15) | 49 (14) | 0.5 |
| Female, n (%) | 145 (78) | 64 (79) | 0.9 |
| FDR with RA, n (%) | 48 (27) | 20 (27) | 1.0 |
| Ever smoker, n (%) | 101 (57) | 47 (59) | 0.8 |
| Current smoker, n (%) | 23 (13) | 14 (18) | 0.3 |
| Follow-up duration in months | 50 (33-62) | 38 (14-59) | **0.04** |
| Symptom duration in months | 21 (10-51) | 20 (10-46) | 0.9 |
| BMI | 25 (22-27) | 25 (23-27) | 0.9 |
| ESR mm/h | 11 (5-19) | 11 (5-19) | 0.8 |
| General health, VAS | 30 (9-52) | 26 (2-49) | 0.9 |
| Pain, VAS | 27 (10-52) | 29 (9-53) | 0.8 |
| RF positive, n (%) | 33 (18) | 54 (67) | **<0.0001** |
| IgG Anti-CCP, AU/ml | 5 (2-29) | 81(20-100) | **<0.0001** |
| HLA-SE carriers (≥1 allele), n (%) | 107 (61) | 58 (73) | 0.1 |

Numbers are presented as median with IQR, interquartile range 1st-3^rd^ if nothing else specified. Abbreviations: N, number; IQR, interquartile range 1st-3rd; FDR, first degree relative (parents, siblings and/or offspring); RA, rheumatoid arthritis; BMI, body mass index; ESR, erythrocyte sedimentation rate; SD, standard deviation; VAS, visual analog scale; RF, rheumatoid factor; HLA-SE, human leukocyte antigen shared epitope. Anti-CCP level, cut off =1; the level is presented as number times increased to a cut off at 1, and with 100 as the upper limit of detection for the Anti-CCP2 assays used. Missing data for FDR, ever smoker, BMI, ESR, VAS general health, VAS pain, HLA-SE: 12/11/45/17/34/34/13 respectively.
